# Safety of interhospital transfer for critically ill COVID-19 patients

**DOI:** 10.1186/s13054-023-04735-9

**Published:** 2023-11-23

**Authors:** Fabian Perschinka, Helmut Niedermoser, Andreas Peer, Georg Franz Lehner, Timo Mayerhöfer, Viktor Stöllnberger, Dietmar Fries, Michael Joannidis, Collaborators, Romuald Bellmann, Adelheid Ditlbacher, Walter Hasibeder, Christoph Krismer, Hannah Antretter, Julia Killian, Stephan Eschertzhuber, Stefanie Zagitzer-Hofer, Eva Foidl, Isabella Weilguni, Stefanie Haslauer-Mariacher, Alexandra Ribitsch, Andreas Mayr, Eugen Ladner, Bernhard Mayr-Hueber, Birgit Stögermüller, Lukas Kirchmair, Bruno Reitter, Miriam Potocnik, Simon Mathis, Anna Fiala, Jürgen Brunner, Claudius Thomé

**Affiliations:** 1 Division of Intensive Care and Emergency Medicine, Department of Internal Medicine, Medical University Innsbruck, Anichstrasse 35, 6020 Innsbruck, Austria; 2 mICU-Alps, Hall, Austria; 3 Mobile Intensiv Medizin, Mils, Austria; 4 Department of General and Surgical Intensive Care Medicine, Medical University Innsbruck, Innsbruck, Austria

During the COVID-19 pandemic, patients had to undergo interhospital transfer (IHT) for multiple reasons such as local shortages or requirement of advanced therapies. The aim of this study was to investigate the safety of ground-based IHT in critically ill COVID-19 patients performed by specifically trained experts.

This retrospective multicentre investigation includes eight hospitals from 1st February 2020 to 14th December 2022. Relocations were considered, when admitting ward was an ICU. All transfers were ground-based conducted by three specialists, with each transport being supervised entirely by one physician (trained for anaesthesia and intensive care) and a medically trained assistance. The PaO_2_/FiO_2_ ratios were obtained in the units before transfer and immediately after admission to the destination ward.

Continuous variables were tested by T test (normal distributed) or Mann-Whitney-U test (not-normal distributed), presented as median with interquartile range. χ^2^ test was used to analyse categorical variables, provided as numbers with corresponding percentages. Correlations were determined by the Pearson correlation coefficient (PaO_2_/FiO_2_ correlations) or the Spearman's rank correlation coefficient (survival correlations).

For comparison between transferred patients and non-relocated patients, a propensity score matching (PSM) (caliper width:  0.01) was applied. PSM included sex, age, IMV, prone positioning, vasopressors, RRT and the number of comorbidities.

We obtained transport data of 175 transfers in 148 patients. The median distance was 70 km (23–95) and the median duration was 50 min (30–85). The transferred cohort was mainly male (66.9%), 62 years (55–73) old and exhibits a SAPS III of 54 (49–63). Additional baseline characteristics of transferred patients and a propensity score matched control cohort are presented in Additional file 1: Table S1.

**Table 1:** Patient transfer characteristics and PaO_2_/FiO_2_ alterations

Transfer characteristics			*n* = 175	
ARDS stage*	No ARDS		9 (6.8%)	
	Mild ARDS		18 (13.6%)	
	Moderate ARDS		50 (37.9%)	
	Severe ARDS		55 (41.7%)	
Distance°		Kilometres	70 (23–95)	
Duration°		Minutes	50 (30–85)	
Positioning*	Supine		167 (95.4%)	
	Prone positioning		8 (4.6%)	
Intubation*	Not endotracheal intubated		72 (41.1%)	
	Endotracheal intubated		98 (56.0%)	
	Ventilation per tracheostoma		5 (2.9%)	
Respiratory support*	BIPAP		64 (36.6%)	
	P_max_°	mbar	26 (24–28)	
	PEEP°	mbar	13 (11–14)	
	FiO_2_°	%	73 (50–90)	
	SIMV		20 (11.4%)	
	P_max_°	mbar	28 (25–30)	
	PEEP°	mbar	14 (13–15)	
	FiO_2_°	%	80 (50–90)	
	Pressure controlled		6 (3.4%)	
	P_max_°	mbar	26 (23–26)	
	PEEP°	mbar	14 (12–15)	
	FiO_2_°	%	60 (40–95)	
	CPAP + pressure support		26 (14.9%)	
	P_max_°	mbar	10 (10–10)	
	PEEP°	mbar	8 (8–10)	
	FiO_2_°	%	60 (45–70)	
	CPAP		23 (13.1%)	
	PEEP°	mbar	8 (8–10)	
	High flow oxygen		4 (2.3%)	
		L/min	16 (16–21)	
	Oxygen mask		32 (18.3%)	
		L/min	10 (4–12)	
ECMO*			6 (3.4%)	
Vasopressors*			76 (43.4%)	
iNO*			4 (2.3%)	
Mortality during transfer*			0	

PaO_2_/FiO_2_ alteration during transfer (mmHg)				*p*
Total cohort (*n* = 143)°	Before transfer	111.0 (80.0–174.9)		0.300
	After transfer	124.4 (85.8–195.0)		
ARDS 2/3 (*n* = 104)°	Before transfer	100.0 (73.5–135.0)		0.031
	After transfer	108.0 (79.2–146.1)		
Death during ICU stay (*n* = 39)°	Before transfer	104.0 (72.0–140.0)		0.643
	After transfer	89.0 (66.7–141.0)		

ARDS—acute respiratory distress syndrome, BIPAP—biphasic positive airway pressure, SIMV—synchronised intermittent mechanical ventilation, CPAP—continuous positive airway pressure, iNO—inhaled nitric oxide

*Number (%); ° median (IQR)

The majority of patients (41.7%) was suffering from severe acute respiratory distress syndrome (ARDS) at begin of transfer [1]. A total of 103 patients (*n* = 58.9%) were invasively mechanically ventilated during the entire transfer.

In the overall cohort, the median PaO_2_/FiO_2_ ratio obtained before start of transport was 111.0 mmHg (80.0–174.9) and 124.4 mmHg (85.8–195.0, *p* = 0.300) after relocation. In the subgroup of patients with moderate or severe ARDS, a statistically significant increase in the PaO_2_/FiO_2_ ratios was observed (100.0 vs. 108.0; *p* = 0.031). No correlation between the changes of PaO_2_/FiO_2_ ratios and duration or distance of transfers (distance: Pearson correlation: 0.086, *p* = 0.307; duration: Pearson correlation: 0.072, *p* = 0.394) was seen (Table 1). Additionally, no correlation between ICU survival and duration or distance was found (distance: correlation coefficient: 0.137, *p* = 0.071; duration: correlation coefficient: 0.125, *p* = 0.099). None of the transferred patients died during transport or within 24 h after transfer.

When comparing transferred patients to a PSM group which was not transferred both, ICU mortality (29.1% vs. 25.0%; *p* = 0.432) and hospital mortality (31.8% vs. 27.0%; *p* = 0.372) tended to be numerically higher. The same pattern was found for ICU and hospital length of stay (Additional file 1: Table S1).

Our analyses showed stable or rising values in PaO_2_/FiO_2_ ratios during transfer. These results are in contrast to previous investigations using air transport [2], which could be caused by a PaO_2_ drop in high altitude or the higher number of hand overs in the mentioned study. Neither the duration nor the distance of the IHTs affected the changes in PaO_2_/FiO_2_ ratios in our analysis, whereas the use of respirators commonly used in ICUs during transport could be one reason for the stable PaO_2_/FiO_2_ ratios. Additionally, the intensified monitoring and ventilatory support during transport may have resulted in better and more personalised care. The physician-to-patient ratio of 1:1 enabled a rapid response to deterioration of patients’ clinical status by adaptation of therapy. This may have a particular effect in moderate to severe ARDS patients and thus lead to a significant improvement. Though remarkable, the even higher PaO_2_/FiO_2_ ratios after transfer in patients with moderate or severe ARDS should be interpreted with caution, as it is unknown whether the improvement of respiratory status was sustainable.

The mortality rates were similar to previous analyses about relocations due to capacity or medical reasons [3, 4]. Higher rates of interventions may reflect a more severe disease state in our transferred patients compared to similar cohorts [5]. However, the fact that no deaths occurred 24 h following the transport indicates that a causal relationship between the transport itself and mortality is unlikely.

The strengths of this study are the comprehensiveness of our registry capturing all critically ill COVID-19 patients from the region during the observation period, the small number of experts having conducted all transfers and recorded PaO_2_/FiO_2_ ratios as respiratory marker. Additionally, the performed PSM (calliper width: 0.01) is precise and enables good comparability. We are aware of some limitations, which include the retrospective character and limited availability of data about administered drugs during IHTs.

This study shows that ground-based IHT can be performed safely and without deterioration of respiratory status by specialised personnel and equipment during a pandemic. These data support using ground-based IHTs as a fixed component of ICU resource optimisation in times of shortages.

### Supplementary Information

**Additional file 1. Table S1:** Comparison between propensity score matched group (critically ill, no transfer) and transferred group.

## Data Availability

The datasets used and analysed in the current study are available from the corresponding author on reasonable request.
