# Supplementary material for: Safety of interhospital transfer for critically ill COVID-19 patients
Source: Crit Care. 2023 Nov 23;27:456. doi: 10.1186/s13054-023-04735-9 (PMC10666401; doi:10.1186/s13054-023-04735-9)
Supplement: Supplementary file 1 — Additional file 1. Table S1: Comparison between propensity score matched group (critically ill, no transfer) and transferred group. [file 13054_2023_4735_MOESM1_ESM.docx]

Electronic supplemental material

**Safety of interhospital transfer for critically ill COVID-19 patients**

**ESM Table 1:** Comparison between propensity score matched group (critically ill, no transfer) and transferred group

|  |  | Control group (no transfer)  (n = 148) | Transferred group  (n = 148) | p |
| --- | --- | --- | --- | --- |
| Sex* | Male | 104 (70.3%) | 99 (66.9%) | .531 |
| Age° |  | 64 (56 – 73) | 62 (55 – 73) | .711 |
| SOFA score° |  | 6 (4 – 9) | 5 (4 – 7) | .047 |
| SAPS III° |  | 55 (46 – 62) | 54 (49 – 63) | .840 |
|  |  |  |  |  |
| **Comorbidities** |  |  |  |  |
| No comorbidities* |  | 22 (15.3%) | 25 (17.4%) | .632 |
| Cardiovascular*** |  | 49 (33.1%) | 49 (33.1%) | 1.000 |
| Hypertension*** |  | 83 (56.1%) | 81 (54.7%) | .815 |
| Diabetes mellitus*** |  | 39 (26.4%) | 32 (21.6%) |  |
| HbA1c%*°* |  | 6.1 (5.7 – 6.6) | 6.2 (5.9 – 6.8) | .165 |
| Renal*** |  | 22 (14.9%) | 25 (16.9%) | .633 |
| Liver*** |  | 9 (6.1%) | 16 (10.8%) | .143 |
| Metastatic*** |  | 2 (1.4%) | 1 (0.7%) | .562 |
| Hematological cancer*** |  | 6 (4.1%) | 7 (4.7%) | .777 |
| Solid cancer*** |  | 8 (5.4%) | 4 (2.7%) | .238 |
| Immunosuppression*** |  | 12 (8.1%) | 12 (8.1%) | 1.000 |
| COPD*** |  | 18 (12.2%) | 14 (9.5%) | .454 |
| Asthma*** |  | 4 (2.7%) | 8 (5.4%) | .238 |
| Respiratory*** |  | 12 (8.1%) | 13 (8.8%) | .834 |
|  |  |  |  |  |
| **Treatment** |  |  |  |  |
| IMV* |  | 122 (82.4%) | 119 (80.4%) | .654 |
| Prone positioning* |  | 115 (77.7%) | 116 (78.4%) | .888 |
| Vasopressors* |  | 120 (81.1%) | 121 (81.8%) | .881 |
| Corticosteroids* |  | 119 (83.8%) | 131 (88.5%) | .245 |
| RRT* |  | 25 (16.9%) | 27 (18.2%) | .760 |
| ECMO* |  | 5 (3.4%) | 25 (16.9%) | <.001 |
| Days IMV° |  | 13 (8 – 21) | 20 (10 – 30) | .003 |
| Days NIV° |  | 3 (1 – 7) | 2 (1 – 6) | .186 |
| Days NHF° |  | 3 (1 – 6) | 4 (1 – 7) | .272 |
| Days prone positioning° |  | 4 (2 – 6) | 4 (2 – 8) | .033 |
| Days RRT° |  | 12 (3 – 26) | 11 (3 – 24) | .640 |
| Days ECMO° |  | 11 (9 – 15) | 26 (15 – 32) | .169 |
|  |  |  |  |  |
| **Outcome** |  |  |  |  |
| ICU mortality* |  | 37 (25.0%) | 43 (29.1%) | .432 |
| Hospital mortality* |  | 40 (27.0%) | 47 (31.8%) | .372 |
| Length of stay ICU° |  | 19 (10 – 28) | 21 (12 – 34) | .123 |
| Length of stay hospital° |  | 27 (18 – 45) | 33 (20 – 47) | .137 |
|  |  |  |  |  |
| **Treatment restrictions** |  |  |  |  |
| No CPR* |  | 22 (14.9%) | 25 (16.9%) | .633 |
| No intervention* |  | 5 (3.4%) | 2 (1.4%) | .251 |
| No ECMO* |  | 22 (14.9%) | 26 (17.6%) | .528 |
| Best supportive care* |  | 38 (25.9%) | 28 (18.9%) | .153 |

** number (%); ° median (IQR)*

*SOFA – sequential organ failure assessment, SAPS III – Simplified Acute Physiology Score III, DM – diabetes mellitus, COPD – chronic obstructive pulmonary disease, HbA1c% – glycated hemoglobin, IMV – invasive mechanical ventilation, RRT – renal replacement therapy, AKI – acute kidney injury, NIV – non-invasive ventilation, NHF – nasal high flow, ECMO – extracorporeal membrane oxygenation, ICU – intensive care unit, CPR – cardiopulmonary resuscitation*
